# Neuroprotective effects of rosemary extract on white matter of prefrontal cortex in old rats

**DOI:** 10.22038/IJBMS.2023.74168.16117

**Published:** 2024

**Authors:** Mohsen Eslami Farsani, Shahnaz Razavi, Homa Rasoolijazi, Ebrahim Esfandiari, Reihaneh Seyedebrahimi, Shima Ababzadeh

**Affiliations:** 1 Anatomy Department, Faculty of Medicine, Qom University of Medical Sciences, Qom, Iran; 2 Cellular and Molecular Research Center, Qom University of Medical Sciences, Qom, Iran; 3 Department of Anatomical Sciences, School of Medicine, Isfahan University of Medical Sciences, Isfahan, Iran; 4 Department of Anatomy, School of Medicine, Iran University of Medical Sciences, Tehran, Iran; 5 Tissue Engineering and Applied Cell Sciences Department, Faculty of Medicine, Qom University of Medical Sciences, Qom, Iran

**Keywords:** Anti-oxidant, Neuroprotective, Prefrontal cortex, Rosemary extract, White matter

## Abstract

**Objective(s):**

During aging, cerebral structures undergo changes due to oxidative stress. The consumption of some plants seems to improve neurological health. For example, rosemary extract (RE) which is widely used as a flavoring food has anti-inflammatory and anti-oxidant activities. Therefore, we aimed to study the effect of RE on the changes related to the aging process in the prefrontal cortex (PFC).

**Materials and Methods:**

Twenty-four male Wistar rats including young and old were purchased. Each group was divided into two subgroups: vehicle and rosemary (old vehicle (OV), old rosemary (OR), young vehicle (YV), and young rosemary (YR) groups). Then, we examined the number of intact neurons, myelin base protein (MBP), white matter (WM), levels of malondialdehyde (MDA), and glutathione peroxidase (GPx) in the PFC.

**Results:**

The results showed that in the old vehicle rats compared to the young group without treatment, except for the MDA level (which increased), other variables significantly decreased (*P*≤0.05). Additionally, RE consumption demonstrated a significant elevation of WMA, MBP intensity, number of intact neurons, and GPx activity level, while MDA levels significantly reduced in the treated old rats compared to the old vehicle group (*P*≤0.05). However, there was no significant difference between the OR and YV groups (*P*≥0.05).

**Conclusion:**

Overall, it seems that RE can protect and improve aging damages in the PFC due to its anti-oxidant properties. So, the use of RE can be a suitable strategy to prevent aging complications in the brain.

## Introduction

Aging has been understood as the collective process of varied harmful changes in the cells and tissues, which is a result of both environmental conditions and genetics (1). Anatomical and functional evidence of the cerebral structures revealed the rate of atrophy changes related to age, depending on the brain region (2), and generally, it happens in white matter (WM) of the temporal and prefrontal cortex (PFC) (3). To explain the aging process, there are numerous hypotheses (4). One of the most important theories is the oxidative stress (OS) hypothesis, which can explain the aging process and other events associated with aging (5). The cells produce reactive oxygen and nitrogen species (ROS/RNS) through various metabolic processes, and these ROS/RNS may cause oxidative damage. However, the anti-oxidants defend the body against ROS/RNS (6). Oxidative damage particularly affects the brain since it requires a great deal of oxygen and has a relatively weak anti-oxidant defense system, especially in the aging process (7). So, it seems that keeping ROS/RNS production under control and anti-oxidant capacity at a desirable level in the long term can help to lower aging damages. For this reason, consumption of many plants and fruits and natural products containing anti-oxidant combinations such as phenolic acids or phenolic diterpenes of rosmarinus acid or carnosic acid is recommended (8).

Rosemary is one of the traditional medicinal herbs with needle-like, evergreen, and aromatic leaves which grows all over the world, especially in the Mediterranean region (9). It has many useful properties including anti-inflammation, anti-carcinogenic, and antiviral (10). The anti-oxidant properties of rosemary are due to phenolic diterpenes such as carnosic acid, rosmarinus acid, and ursolic acid, and these components act as hydrogen donors in the reaction of free radical molecules (11, 12). 

The effects of rosemary on neurodegenerative diseases showed that this herb improves long-term memory and dementia through prevention of acetylcholinesterase activity and stimulation of butyrylcholinesterase in the rat brain (13). Also, it was proved that consumption of rosemary compounds such as carnosic acid reduces amyloid plaque number and astrogliosis, while it increases synaptic and dendritic markers in Alzheimer’s disease models (14).

Nutritional anti-oxidants may play an important role in preventing the aging process by reducing oxidative damage (15). Therefore, this study aimed to investigate the effect of oral rosemary extract consumption on the glutathione peroxidase (GPx) activity, rate of MDA, structure of histology, and immunohistochemistry of WM of PFC in old and young mice. 

## Materials and Methods

***Materials***

Toluidine Blue (89640), cresyl violet acetate (C1791-5g), and 4′,6-diamidino-2-phenylindole (DAPI) were purchased from Sigma Chemical Co (Saint Louis, Missouri USA). The rosemary extract was purchased from Hunan Geneham Biomedical Technological Company of China and included 40% carnosic acid (RAP20-110401). GPx and MDA kits were obtained from Randox, UK. The anti-myelin basic protein (anti-MBP) and rabbit anti-mouse FITC were supplied by Abcam. 

***Animals***

Twenty-four male Wistar rats were used in this study. The rats were purchased from the animal laboratory at Isfahan University of Medical Sciences and were maintained under a 12 hr light/dark cycle and a constant temperature of (21 ± 2 °C) with free access to food and water. This study was approved by the Ethics Committee of the Isfahan University of Medical Sciences (Grant no. 393236). 

The rats were divided into old (18 months) and young (2 months) groups. Each group consisted of sub-groups: old vehicle (OV, n=6), old rosemary (OR, n=6) groups, young vehicle (YV, n=6), and young rosemary (YR, n=6) groups. The animals in the vehicle groups received 1 ml of distilled water orally via animal-feeding incubation needles (Perfecta, German), while the animals in the rosemary groups received rosemary extract (100 mg/kg/day, orally) dissolved in 1 ml of distilled water for 12 weeks based on the previous studies (16, 17). After treatment, the rats were anesthetized using ketamine and xylazine (150 mg/kg and 15 mg/kg, respectively) and were perfused through the left cardiac ventricle with 200 ml of saline solution, followed by 200 ml of paraformaldehyde 4% in 0.1 M phosphate buffer saline (PBS). Then, samples of the brain were fixed with paraformaldehyde 4% overnight, cleared in xylene, and embedded in paraffin. The brains were cut by a rotary microtome (LEICA RM 2235) and then stained with Toluidine blue or Cresyl-violet (Nissl staining). Serial sections were prepared (1.3–4.3 mm of Bregma) from the PFC region at a thickness of 4 µm in the coronal plane of each brain. The five sections of each brain were used for each histology technique.

***Toluidine blue stains***

Toluidine blue is a synthetic, acidophilic, metachromatic dye that has an affinity for nucleic acids therefore, it binds to a high DNA and RNA content in chromatin or Nissl substance and selectively stains the nucleus as dark blue and the cytoplasm as light blue. After sectioning, slides were immersed in a solution of 1% KMno4 for 1-5 min. Then, the slides were rinsed with distilled water and bleached with 5% oxalic acid. 1% toluidine blue solution was used for staining and slides were dipped in 0.2% uranium nitrate for a few seconds. Finally, the slides were observed by a light microscope and photomicrographs were studied using Image-j software for analyzing the WM (18).

***Cresyl*** *-* ***violet staining (Nissl staining)***

PFC sections were stained with 0.1% cresyl violet for 30 min at 56 °C to demonstrate intact cell bodies. To this end, five sections of each PFC were analyzed by a microscope at 40X magnification. The intact cells were counted in the four fields using Image-j software. Finally, the average cell number of five sections was considered as the cell number of each rat (19).

***Immunohistochemistry***

The immunohistochemical staining for MBP was performed on formalin-fixed and paraffin-embedded brain tissue. The brain sections were treated with HCl at 37 °C for 10 min. The endogenous peroxidase activity was inhibited using 3% hydrogen peroxide for 10 min. Then, the sections were washed with PBS and incubated in a blocking solution (PBS containing 3% goat serum and 0.2% TritonX-100) for 30 min at room temperature. After washing with PBS, the sections were incubated overnight at 4 °C with anti-myelin basic protein antibody (MBP101) (ab62631) diluted in PBS (1:1000) (20). Afterward, the sections were washed and incubated with goat anti-mouse IgG H& L (FITC) (ab6785) (1:400) (21). The cell nuclei were stained with DAPI (1:1000). The stained sections were studied under a fluorescent microscope (Olympus BX51, Japan). 

***Anti-oxidant enzymes detect***

After the treatment, the remaining rats were anesthetized with ketamine (150 mg/kg) and xylazine (15 mg/kg). The brains were immediately removed and the PFC was isolated. Then, the samples were immersed in liquid nitrogen and stored at −80 °C until biochemical analysis. Afterward, the tissue samples were homogenized in ice-cold Tris-Hcl (1:10 W/V) and then centrifuged at 12000 ×g for 20 min at 4 °C. The supernatants were separated and used for a GPx enzyme activity assay and MDA. 

***GPx and MDA activity***

GPx activity was determined spectrophotometrically based on the Wheeler method (22) and using Randox kit recommendations. The reaction was initiated by adding 0.2 mm of H_2_O_2_, 0.1 mm of GSH, 0.14 U of glutathione reductase, 1.5 mm of NADPH, 0.1 mm of sodium acid, 0.1 M phosphate buffer, and 1 mg/ml of the hippocampus supernatant at room temperature. The enzyme activity was calculated based on oxidized nicotinamide adenine dinucleotide phosphate. The absorbance changes were recorded at 340 nm. MDA is used as a standard measurement for oxidation level damage by free radicals and this process was characterized by the Satoh method (16). 

***Statistical analysis***

The data were presented as the means ± standard error and analyzed by the SPSS statistical software, version 26. Statistical comparisons between groups were performed through one-way analysis of variance (ANOVA), followed by Tukey’s *post hoc* test, where the *P*-value<0.05 was considered statistically significant.

## Results

***The white matter area***

**Figure 1:**
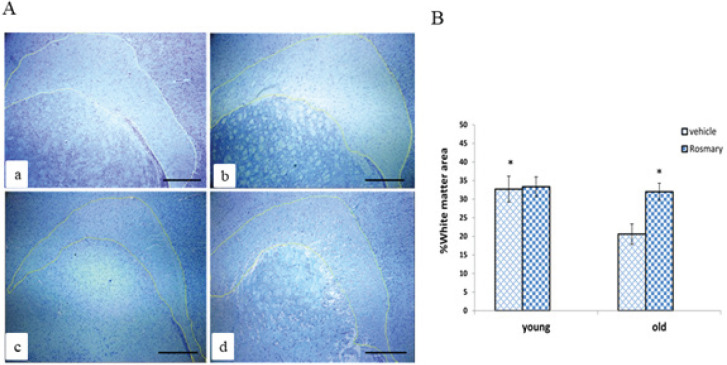
(A) Representative images of the white matter area following Toluidine blue staining ×20, Scale bar=200 µm (a–d); The young vehicle (YV) group (a), young rosemary (YR) group (b), old vehicle (OV) group (c), old rosemary (OR) group (d). (B) Comparison of the mean percentage of the white matter (WM) area in different groups (mean ± SEM)

The results of the staining of PFC revealed a significant decrease in the percentage of the WM area in the OV group compared with the YV group (*P*≤0.05). As shown in Figure 1, a significant increase in the mean percentage of the WM area was observed in the OR group compared with the OV group (*P*≤0.05). However, there were no significant differences between neither OR and YV groups nor the YV and YR groups (*P*≥0.05).

***Intact cell count***

**Figure 2:**
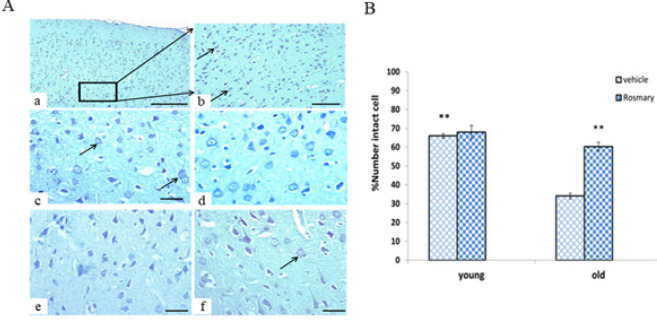
(A) Photomicrograph shows the intact cells (arrows) in the medial prefrontal cortex (PFC) in different groups (a: young vehicle (YV) with X10, b: YV with 20X, and c, d, e, f related to YV, young rosemary (YR), old vehicle (OV), old rosemary (OR) groups, respectively with 40X magnification). Scale =500 µm. (B) Histogram shows differences in the mean intact cells in different groups (mean ± SEM)

As displayed in Figures 2A and B, a significant reduction was observed in the mean percentage of intact cells of PFC in the old rats without treatment compared to the normal young rats (*P*≤0.01). Also, treatment with rosemary extract in old rats significantly increased the percentage of intact cells of the PFC compared to the OV group (*P*≤0.01).

***Percent of MBP density***

**Figure 3:**
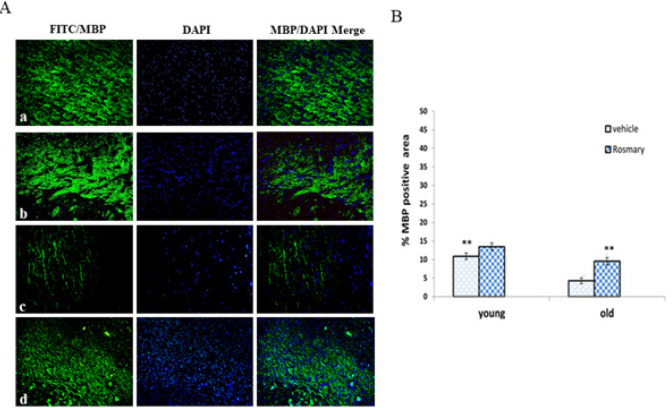
(A) IHC photomicrograph of myelin base protein (MBP) antibody and 4′,6-diamidino-2-phenylindole (DAPI) staining in different groups: young vehicle (YV) group (a), young rosemary (YR) group (b), old vehicle (OV) group (c), and old rosemary (OR) group (d), X40 magnification, scale bar=500 µm. (B) The comparison of the mean percentage of MBP positive cells in different groups (mean ± SEM)

The treatment efficiency of rosemary extract was evaluated by immunocytochemistry analysis (Figure 3A) and the cells of the PFC region were stained with the marker against MBP (myelinating potential), and cell nuclei were stained with DAPI. The data demonstrated a significant decrease in the mean percentage of MBP density in the OV group compared to the YV group (*P*≤0.01) and a significant increase in the OR group compared to the OV group (*P*≤0.01). However, the results show no significant difference between old rats treated with rosemary extract and young rats without treatment (*P*≥0.05) (Figure 3B).

***Anti-oxidant enzyme activity in the PFC***

**Figure 4:**
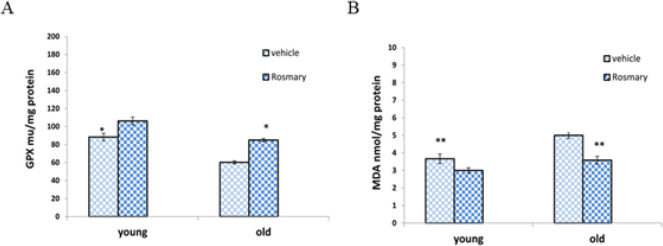
(A) Comparison of the mean level of glutathione peroxidase (GPx) in different groups, (B) Comparison of the mean level of malondialdehyde (MDA) in various groups (mean ± SEM)

Figure 4A shows that GPx enzyme activity level in the PFC was significantly decreased in the OV group compared to the YV group (*P*≤0.01). However, a significant increase of GPx activity level was observed after treatment with rosemary extract in old rats compared to the OV group (*P*≤0. 01). There was no significant difference in the GPx activity level between treated groups with rosemary extract and young rats without treatment (*P*≥0.05). 

***Determination of MDA concentration***

The concentration of MDA was measured as a ROS marker in the PFC (Figure 4B). Although it was noticed that the mean MDA level significantly decreased in treated old rats compared to old rats without treatment (*P*≤0.05), there was no significant difference between the treated groups and the YV group (*P*≥0.05).

(* *P*<0.05: significant difference between OV group with different groups), the number of animals (n=6) in each group.

(** indicates significant difference at *P*<0.01 between the OV group and other groups), the number of animals (n=6) in each group.

(** °C1: significant difference between OV with different groups), the number of animals (n=6) in each group.

(* °C5 and ** °C1 indicate significant difference between the OV group with different groups), the number of animals (n=6) in each group.

## Discussion

The present study indicated a significant decline in GPx activity level and a significant increase of MDA in the OV group. In contrast, the three-month RE diet caused a significant increase in GPX level and a significant decrease in MDA level in the treated old rats compared to the OV group. 

GPx is one of the important enzymatic anti-oxidants that prevent oxidative damage (23) and there is an inverse relationship between age and plasma GPx activities (24), while MDA is a lipid oxidation end product and can be applied as an important biomarker of oxidative stress (25). Also, there is a controversial relationship between GPx anti-oxidants and MDA levels (26). In the hippocampus of aging rats, consumption of RE (100 and 50 mg/kg) led to a significant increase in the GPx activity level (16). In fact, the reaction between polyunsaturated lipids and ROS / RNS leads to MDA production during the aging process, which in turn reflects elevated MDA levels.

This suggests the positive anti-oxidant effect of RE as a preventative agent in lipid peroxidation during the aging process. It seems that RE and its anti-oxidant compounds inhibit lipid peroxidation and free radical production both *in vitro* and *in vivo* (27). These results suggest that RE is likely to prevent and reduce ROS/RNS and inhibit oxidative damage during aging thanks to containing many anti-oxidants, anti-inflammatory, and anti-cancer compounds. For example, carnosic acid is a lipophilic anti-oxidant that scavenges free radicals. Therefore, carnosic acid can prevent lipid peroxidation and protect biological membranes (28). A similar study suggested, that the use of rosemary and *Bacopa monnieri* caused an increase in anti-oxidant properties, reduced lipid peroxidation, increased the production of brain-derived neurotrophic factors, and inhibited the production of amyloid precursor proteins in the glial cell and embryonic mouse hypothalamus cells (29). The polyphenol content is one of the other compounds of RE that plays an important role in absorbing and neutralizing free radicals, quenching singlet and triplet oxygen, and decomposing peroxides (30). Also, RE consumption reduced lipid peroxidation and ROS levels in the cerebrum of aged rats (31). It has been reported that treatment with rosemary leaf extract (100 mg/kg) for 3 weeks caused a significant increase in epinephrine levels in the cerebral cortex (32). In fact, rosemary and its derivatives can improve age-related changes in the brain through several mechanisms such as the greater production of nerve growth factors, total choline level, acetylcholine, decrease of atrophy of cholinergic neurons and hippocampal neurons against Aβ, and inhibition of acetylcholinesterase and butyrylcholinesterase in the brain (33). 

Furthermore, the results of this study demonstrated a significant decrease in the mean percentage of rat PFC intact cells in the OV group compared to the YV group. Some studies have reported that ROS/RNSs such as O_2_^-^, OH, H_2_O_2_, and nitric oxide (NO) are created in all aerobic cells and generally exist in equilibrium with the anti-oxidant system (34, 35). The aging process reflects the accumulation of free radicals in cells and tissues (36). Commonly, neuronal losses occur following aging in the human brain even if its volume appears normal (37). Thus, the loss of intact cells in PFC following aging can be due to the increase of oxidative damages (ROS/RNS) and the decline of the anti-oxidant defense system or loss of balance between them (38). In the present study, the administration of RE in old rats increased intact cells in PFC due to RE improvement of the anti-oxidant system and free radical scavenging, resulting in the protection of neurons against oxidative damage and reduction of cell injury.

We observed a reduction in the percentage of the WM area in the OV group compared with the YV group, while an increase was observed in treated old rats, which was not significantly different from the YV group. WM occupies a wide volume of the human cerebrum and it includes myelinated axons and many myelin-producing glial cells. In aging, PFC particularly WM in this region undergoes fundamental changes, and its thickness decreases about 5% every 10 years (39). Studies based on MRI evaluations emphasize that WM volume decreases given the age of the monkey and human brain, mainly in the frontal lobe (40, 41). Also, a study showed that the integrity of WM structure in the normal aging process was less organized compared to the young (42). Generally, cortical thinning especially in the frontal and temporal regions has been found following aging (43). 

The MBP density of PFC was evaluated by the antibody against MBP and IHC technique in the OR group. This protein is one of the most important proteins of myelin commonly used as a marker for myelin expression. MBP forms almost 30% of all myelin proteins and consists of two families: classic- and golli-MBP (44, 45). Classic-MBP is found in mature oligodendrocytes and myelin sheaths (46). 

In general, immunohistochemically staining, labeling the Classic-MBP utilized for myelin staining (7). As previously mentioned, our data MBP intensity showed a significant decrease in the OV group, while there was no significant difference between treated old rats with RE compared to young rats without treatment.

## Conclusion

The main results of this study reveal that the normal aging process can cause structural and functional deteriorating changes in rat’s PFC (WMA, myelinated fibers, anti-oxidant capacity, lipid peroxidation, and cortical cell). However, a three-month consumption of RE can have beneficial effects on preventing and improving these changes.

## Authors’ Contributions

S R designed the experiments; M EF, H R, and E E performed experiments and collected data; M EF and H R discussed the results and strategy; S R Supervised, directed, and managed the study; all authors approved the final version to be published.

## Conflicts of Interest

The authors have no conflicts of interest to declare.

## References

[1] Rodríguez-Rodero S, Fernández-Morera JL, Menéndez-Torre E, Calvanese V, Fernández AF, Fraga MF (2011). Aging genetics and aging. Aging Dis.

[2] Lockhart SN, DeCarli C (2014). Structural imaging measures of brain aging. Neuropsychol Rev.

[3] Fjell AM, McEvoy L, Holland D, Dale AM, Walhovd KB, Initiative AsDN (2013). Brain changes in older adults at very low risk for Alzheimer’s disease. J Neurosci.

[4] Cefalu CA (2011). Theories and mechanisms of aging. Clin Geriat Med.

[5] Harman D (2009). Origin and evolution of the free radical theory of aging: A brief personal history, 1954–2009. Biogerontology.

[6] Poljšak B, Fink R (2014). The protective role of anti-oxidants in the defence against ROS/RNS-mediated environmental pollution. Oxid Med Cell Longev.

[7] Posadas S, Caz V, Largo C, De la Gandara B, Matallanas B, Reglero G (2009). Protective effect of supercritical fluid rosemary extract, Rosmarinus officinalis, on anti-oxidants of major organs of aged rats. Exp Gerontol.

[8] Kelsey NA, Wilkins HM, Linseman DA (2010). Nutraceutical anti-oxidants as novel neuroprotective agents. Molecules.

[9] Abd El-Ghany MA, Motawee M (2012). Biological effects of yoghurt with rosemary on injured liver rats. Aust J Basic Appl Sci.

[10] Ojeda-Sana AM, van Baren CM, Elechosa MA, Juárez MA, Moreno S (2013). New insights into antibacterial and anti-oxidant activities of rosemary essential oils and their main components. Food Control.

[11] Doolaege EH, Vossen E, Raes K, De Meulenaer B, Verhé R, Paelinck H (2012). Effect of rosemary extract dose on lipid oxidation, colour stability and anti-oxidant concentrations, in reduced nitrite liver pâtés. Meat Sci.

[12] Rahbardar MG, Hosseinzadeh H (2020). Therapeutic effects of rosemary (Rosmarinus officinalis L ) and its active constituents on nervous system disorders. Iran J Basic Med Sci.

[13] Ozarowski M, Mikolajczak PL, Bogacz A, Gryszczynska A, Kujawska M, Jodynis-Liebert J (2013). Rosmarinus officinalis L leaf extract improves memory impairment and affects acetylcholinesterase and butyrylcholinesterase activities in rat brain. Fitoterapia.

[14] Lipton SA, Rezaie T, Nutter A, Lopez KM, Parker J, Kosaka K (2016). Therapeutic advantage of pro-electrophilic drugs to activate the Nrf2/ARE pathway in Alzheimer’s disease models. Cell Death Dis.

[15] Simioni C, Zauli G, Martelli AM, Vitale M, Sacchetti G, Gonelli A (2018). Oxidative stress: role of physical exercise and anti-oxidant nutraceuticals in adulthood and aging. Oncotarget.

[16] Rasoolijazi H, Mehdizadeh M, Soleimani M, Nikbakhte F, Farsani ME, Ababzadeh S (2015). The effect of rosemary extract on spatial memory, learning and anti-oxidant enzymes activities in the hippocampus of middle-aged rats. Med J Islam Repub Iran.

[17] Pourbabaki R, Khadem M, Samiei S, Hasanpour H, Shahtaheri SJ (2020). The protective effect of rosemary in mitigating oxidative stress induced by Chlorpyrifos in rat kidney. J Health Saf Work.

[18] Farsani M, Dakhili M, Ababzadeh S, Yeganehparast M, Heidari F (2019). Histomorphological and histochemical effects of diet with Qom zeolite on the tissue structure of the small intestine of broiler chickens compared with commercial zeolite. J Vet Res.

[19] Hamidizad Z, Ababzadeh S, Heidari F, Haeri N-a-S, Eslami Farsani M, Sadegh M (2019). Cobalamin modulate neurotoxic effects of trimethyltin chloride on hippocampus neural cells and cognitive function. Physiol Pharmacol.

[20] Zhang X-Q, Wang X-Y, Dong B-C, Li M-X, Wang Y, Xiao T (2023). CXC chemokine receptor type 7 antibody enhances neural plasticity after ischemic stroke. Neural Regen Res.

[21] Ding L, Hao K, Sang L, Shen X, Zhang C, Fu D (2023). ATF2-driven osteogenic activity of enoxaparin sodium-loaded polymethylmethacrylate bone cement in femoral defect regeneration. J Orthop Surg Res.

[22] Rubiolo JA, Mithieux G, Vega FV (2008). Resveratrol protects primary rat hepatocytes against oxidative stress damage:: Activation of the Nrf2 transcription factor and augmented activities of anti-oxidant enzymes. Eur J Pharmacol.

[23] Maes M, Mihaylova I, Kubera M, Uytterhoeven M, Vrydags N, Bosmans E (2010). Lower whole blood glutathione peroxidase (GPX) activity in depression, but not in myalgic encephalomyelitis/chronic fatigue syndrome: another pathway that may be associated with coronary artery disease and neuroprogression in depression. Neuro Endocrinol Lett.

[24] Amirkhizi F, Siassi F, Djalali M, Foroushani AR (2010). Assessment of anti-oxidant enzyme activities in erythrocytes of pre-hypertensive and hypertensive women. J Res Med Sci.

[25] Marrocco I, Altieri F, Peluso I (2017). Measurement and clinical significance of biomarkers of oxidative stress in humans. Oxid Med Cell Longev.

[26] Bilgin R, Kurt N, Gulesci N, Yucebilgic G (2020). Age-related changes in anti-oxidant enzyme activities. Ann Clin Nutr.

[27] Al Sheyab FM, Abuharfeil N, Salloum L, Hani RB, Awad DS (2012). The effect of Rosemary (Rosmarinus officinalis ) plant extracts on the immune response and lipid profile in mice. J Biol Life Sci.

[28] Loussouarn M, Krieger-Liszkay A, Svilar L, Bily A, Birtić S, Havaux M (2017). Carnosic acid and carnosol, two major anti-oxidants of rosemary, act through different mechanisms. Plant Physiol.

[29] Ramachandran C, Quirin K-W, Escalon E, Melnick SJ (2014). Improved neuroprotective effects by combining Bacopa monnieri and Rosmarinus officinalis supercritical CO2 extracts. J Evid Based Complementary Altern Med.

[30] Yashin A, Yashin Y, Xia X, Nemzer B (2017). Anti-oxidant activity of spices and their impact on human health: A review. Anti-oxidants.

[31] Posadas S, Caz V, Largo C, De la Gándara B, Matallanas B, Reglero G (2009). Protective effect of supercritical fluid rosemary extract, Rosmarinus officinalis, on anti-oxidants of major organs of aged rats. Exp Gerontol.

[32] Waggas AM, Balawi AE (2008). Neurophysiological study on possible protective effect of rosemary (Rosemarinus officinalis) Leaves Extract in male albino rats treated with acrylamide. Am-Eurasian J Sci Res.

[33] Hussain S, Syeda A, Alshammari M, Alnasser S, Alenzi N, Alanazi S (2022). Cognition enhancing effect of rosemary (Rosmarinus officinalis L ) in lab animal studies: A systematic review and meta-analysis. Braz J Med Biol Res.

[34] Maes M, Galecki P, Chang YS, Berk M (2011). A review on the oxidative and nitrosative stress (O&NS) pathways in major depression and their possible contribution to the (neuro) degenerative processes in that illness. Prog Neuropsychopharmacol Biol Psychiatry.

[35] Suthammarak W, Somerlot BH, Opheim E, Sedensky M, Morgan PG (2013). Novel interactions between mitochondrial superoxide dismutases and the electron transport chain. Aging Cell.

[36] Kumar H, Lim H-W, More SV, Kim B-W, Koppula S, Kim IS (2012). The role of free radicals in the aging brain and Parkinson’s disease: convergence and parallelism. Int J Mol Sci.

[37] Morterá P, Herculano-Houzel S (2012). Age-related neuronal loss in the rat brain starts at the end of adolescence. Front Neuroanat.

[38] Liguori I, Russo G, Curcio F, Bulli G, Aran L, Della-Morte D (2018). Oxidative stress, aging, and diseases. Clin Interv Aging.

[39] Hantke N, Bott NT (2020). Neuropsychology with older adults. Handbook Mental Health Aging.

[40] Shaw ME, Sachdev PS, Anstey KJ, Cherbuin N (2016). Age-related cortical thinning in cognitively healthy individuals in their 60s: the PATH through life study. Neurobiol Aging.

[41] Wisco JJ, Killiany RJ, Guttmann CR, Warfield SK, Moss MB, Rosene DL (2008). An MRI study of age-related white and gray matter volume changes in the rhesus monkey. Neurobiol Aging.

[42] Tian Q, Ferrucci L, Resnick SM, Simonsick EM, Shardell MD, Landman BA (2016). The effect of age and microstructural white matter integrity on lap time variation and fast-paced walking speed. Brain Imaging Behav.

[43] Mohammadi H, Peng K, Kassab A, Nigam A, Bherer L, Lesage F (2021). Cortical thinning is associated with brain pulsatility in older adults: An MRI and NIRS study. Neurobiol Aging.

[44] Harauz G, Ladizhansky V, Boggs JM (2009). Structural polymorphism and multifunctionality of myelin basic protein. Biochemistry.

[45] Harauz G, Boggs JM (2013). Myelin management by the 18 5-kDa and 21 5-kDa classic myelin basic protein isoforms. J Neurochem.

[46] Wake H, Lee PR, Fields RD (2011). Control of local protein synthesis and initial events in myelination by action potentials. Science.

